# Scientific Integrity and Transparency in Academic Writing: The Foundation of Credible Science

**DOI:** 10.3390/children11101191

**Published:** 2024-09-29

**Authors:** Niels Wedderkopp, Erich Rutz

**Affiliations:** 1 Department of Clinical Research, University of Southern Denmark, 5230 Odense, Denmark; 2 The Spine Center of Southern Denmark, 6000 Kolding, Denmark; 3 Depatment of Orthopaedics, The Royal Children’s Hospital, Melbourne 3052, Australia; 4 Murdoch Children’s Research Institute, Melbourne 3052, Australia; 5 Department of Paediatrics, Bob Dickens Chair, Paediatric Orthopaedic Surgery, The University of Melbourne, Melbourne 3010, Australia; 6 Medical Faculty, University of Basel, 4001 Basel, Switzerland

## 1. Introduction

In the academic community, discussions and debates are a natural and vital part of the research process. These discussions drive the continuous refinement and evolution of scientific knowledge. It is important to recognize that, unless research is marred by serious flaws, selective censoring of references, or severe cognitive bias, these debates are inherently beneficial.

## 2. Sound Methodology and Scientific Integrity

At the heart of credible research lies sound methodology and unwavering scientific integrity. Methodology refers to the systematic, theoretical analysis of the methods applied to any field of study. It encompasses concepts such as paradigms, theoretical models, phases, and quantitative or qualitative techniques. By adhering to a robust and well-defined methodology, researchers should ensure that their findings are reliable, valid, and reproducible. This involves the careful planning of the study design, meticulous data collection, appropriate statistical analysis, and transparent reporting of results.

Scientific integrity, on the other hand, pertains to the adherence to ethical principles and professional standards essential for the responsible practice of research. This includes honesty, accuracy, efficiency, and objectivity. Researchers must report their findings truthfully without fabrication, falsification, or inappropriate data manipulation. They also need to give credit where it is due and avoid plagiarism.

## 3. Transparency in Research

Full transparency is a cornerstone of credible research. It involves openness in the entire research process, from the formulation of hypotheses and the study design to the collection and analysis of data and the publication of the results. Transparency allows other researchers to scrutinize and replicate studies, which is essential for the verification and validation of their findings.

Journals play a crucial role in promoting transparency. They can enforce policies that require authors to fully disclose their methodology and data. This includes the sharing of raw data when possible, the publication of detailed protocols, and the acknowledgment of any potential conflicts of interest.

## 4. Benefits of Academic Discussion

Academic discussions, when conducted respectfully and constructively, are beneficial to the research community! These discussions can take the form of critiques, debates, or discussions in academic journals, conferences, and seminars. They allow researchers to:

**Identify and Correct Errors:** Constructive criticism can help identify flaws or gaps in a study that the original researchers may have overlooked. This can lead to corrections and improvements in the research.

**Generate New Ideas:** Debates and discussions can stimulate new ideas and perspectives. They can challenge existing paradigms and lead to the development of new theories and methodologies.

**Enhance Understanding:** Discussions can deepen the understanding of a topic by bringing together diverse viewpoints and expertise. This can be particularly valuable in interdisciplinary research where different fields converge.

**Promote Collaboration:** Engaging in academic discussions can lead to collaborations between researchers. These collaborations can bring together complementary skills and knowledge, leading to more comprehensive and innovative research.

## 5. Avoiding Bias and Ensuring Fairness

To maintain the integrity of academic discussions, it is crucial to avoid selective censoring and cognitive biases. Selective censoring, the practice of only including studies that support a particular viewpoint, undermines the credibility of the research. Cognitive bias, such as confirmation bias, where researchers favor information that confirms their preconceptions, can also skew the research process and outcomes.

To mitigate these issues, researchers and journals should adopt measures such as:

**Pre-Registration:** Researchers should pre-register their study protocols, including hypotheses, methodologies, and analysis plans, before collecting data. This can help prevent selective reporting and p-hacking (manipulating data to find statistically significant results).

**Blinded Peer Review:** To reduce bias, we use single-blinded peer review with at least two independent reviewers, followed by a final decision by the editor responsible for the academic quality of the publication process, including acceptance decisions.

**Conflict of Interest Disclosures:** Researchers should disclose any potential conflicts of interest that could influence their work. Journals should enforce policies to ensure that these disclosures are made transparently.

## 6. The Role of Journals in Promoting Transparency

Journals can play a pivotal role in ensuring that research is conducted and reported transparently. This can be achieved through:

**Clear Guidelines:** Providing clear guidelines for authors on what information needs to be included in their submissions.

**Data Sharing Policies:** Encouraging or requiring authors to share their raw data and code when possible.

**Open Access:** Promoting open access to research findings so that they are available to the wider community, including policymakers, practitioners, and the public.

**Ethical Standards:** Upholding high ethical standards for publication, including the fair and unbiased review of manuscripts.

## 7. Conclusions

In conclusion, discussions around research are essential for the advancement of knowledge. As long as they are free from serious flaws, selective censoring, and cognitive biases, these discussions can significantly benefit the research community. By adhering to sound methodology and scientific integrity and promoting full transparency, journals and researchers can ensure that academic discussions are constructive and lead to meaningful advancements in their respective fields. This not only enhances the quality and reliability of research but also fosters a culture of openness and collaboration that is crucial for scientific progress.

